# Influence of Secondary School Students’ Physical Fitness on Sports Performance during an Ultimate Frisbee Competition

**DOI:** 10.3390/ijerph19073997

**Published:** 2022-03-28

**Authors:** Javier Portillo, Alfredo Bravo-Sánchez, Pablo Abián, Alberto Dorado-Suárez, Javier Abián-Vicén

**Affiliations:** 1 Motor Competence and Excellence in Sport, Faculty of Sport Sciences, University of Castilla-La Mancha, 45071 Toledo, Spain; luis.portillo@uclm.es (J.P.); alberto.dorado@uclm.es (A.D.-S.); 2 Performance and Sport Rehabilitation Laboratory, Faculty of Sport Sciences, University of Castilla-La Mancha, 45071 Toledo, Spain; alfredo.bravo@uclm.es; 3 Faculty of Humanities and Social Sciences, Comillas Pontifical University, 28049 Madrid, Spain; pabloo9@hotmail.com

**Keywords:** high school, alternative sport, ALPHA fitness test, physical education, game analysis

## Abstract

The aim of the present study was to investigate the effect of secondary school students’ fitness profile on physical and technical–tactical performance in simulated competition conditions of ultimate frisbee when there is no previous experience in the practice of the sport. Forty-three secondary school students participated in this research. The students were divided into two groups according to their results in the Assessing Levels of Physical Activity and fitness test battery (ALPHA fitness test): poor physical fitness (PPF) (N = 24; age: 14.9 ± 0.8 years; height: 166.1 ± 10.9 cm; body mass: 62.2 ± 11.0 kg; ALPHA fitness score: 2.7 ± 0.7 points) and good physical fitness (GPF) (N = 19; age: 14.5 ± 0.6 years; height: 165.9 ± 5.8 cm; body mass: 58.9 ± 7.5 kg; ALPHA fitness score: 4.4 ± 0.3 points). Physical variables during the ultimate frisbee match were assessed using Global Positioning System technology. The matches were video-recorded, and individual technical actions were noted afterwards. The GPF group showed higher values for running (*p* = 0.039), high-speed running (*p* = 0.015), sprinting (*p* = 0.022) and total distance covered (*p* = 0.025) than the PPF group. In addition, more passes (*p* = 0.019), offensive decision making (*p* = 0.009) and player participation (*p* = 0.046) were recorded in the GPF group than the PPF group. Correlational analysis revealed a positive relationship (*p* < 0.05) between individual participation and the meters covered for jogging, running, running at high speed and sprinting during the game. In conclusion, although the students were novices in ultimate frisbee, the high physical fitness level had a positive effect on the game performance. Physical education teachers should consider this information when introducing new sports into their physical education classes.

## 1. Introduction

Physical education is the subject in the educational curriculum whose main objective is to promote the physical, mental and socio-emotional well-being of the student through the experimentation of a variety of physical and sporting activities. The practice of continuous physical activity offers several physiological, musculoskeletal or neurobiological benefits for the human organism [1,2,3]. In the school context, different fitness tests are used to assess the physical fitness of the students and to help the teacher identify those students with a high risk of developing health problems (cardiovascular disease, type 2 diabetes or high blood pressure) in the future [4,5,6].

Physical demands in physical education differ according to the nature of the tasks performed during the sessions (i.e., dance, fitness, gymnastics, team sports, individual sports, analytical tasks). To improve schoolchildren’s health, physical education sessions should achieve an adequate intensity range of physical activities. Therefore, it is essential to investigate the factors that facilitate reaching the target intensity of physical activity that contribute to the positive effects of physical education sessions independently of the type of activities that the schoolchildren are practising. According to Fairclough and Stratton [7], the range of moderate-to-vigorous physical activity (MVPA) during the session should represent at least 50% of the session time. Research analysing the physical activity levels of schoolchildren during physical education sessions indicate that the students usually do not meet the recommended target [3].

The type of activity or the student’s motor competence profile have also been analysed in order to clarify the factors involved in the development of health benefits in physical education sessions [7,8,9]. Research in physical education sessions has been conducted under heterogeneous conditions (age of participants, type of activities, socio-cultural environment, protocols and measurement instruments) and most studies have indicated that sessions based on games or team sports activities require a higher pattern of MVPA than sessions focused on the improvement of motor skills or individual games [7,8,9]. In addition, the students with high motor competence profile have significantly higher MVPA physical activity ranges than students with lower levels of ability, so the motor competence profile impacts participation in physical education sessions [6,7,10], although this fact should be analysed when a new sport is introduced.

Ultimate frisbee is a growing team sport around the world, with millions of players in North America, Europe and Asia [11]. The most common team sports taught in physical education session are soccer, basketball and handball, although ultimate frisbee is also growing in popularity in this context [12]. During physical education sessions, the students should be able to express their physiological abilities, technical–tactical skills and decision making in the best possible way [13]. The interaction of physical fitness and motor competence has been investigated in the transition from elementary to high school in which high school adolescents tend to reduce their daily physical activity; physical fitness has been described as a powerful mediator for the results achieved on motor control tests [14,15]. However, the influence of physical fitness on game performance is still an under-researched scenario in the ecology of physical education sessions, even more so when a new sport is introduced. Consequently, more research is needed to show whether physical fitness (speed, muscular strength, agility and maximal aerobic power) affects student performance in team sports [16,17]. Therefore, the purpose of this study was to investigate the effect of the student’s fitness profile on physical and technical-tactical performance in simulated competition conditions when there is no previous experience in the practice of the sport, in this case, ultimate frisbee. We hypothesise that match performance may be positively associated with schoolchildren’s physical fitness.

## 2. Materials and Methods

### 2.1. Participants and General Procedure

Forty-three secondary school students participated in this study. The students came from state schools in the region of Castilla–La Mancha (Spain) and were divided into two groups according to their results in the Assessing Levels of Physical Activity and fitness test battery (ALPHA fitness): poor physical fitness (PPF) (N = 24; age: 14.9 ± 0.8 years; height: 166.1 ± 10.9 cm; body mass: 62.2 ± 11.0 kg; body mass index: 22.5 ± 2.6 arbitrary units (A.U.); ALPHA fitness score: 2.7 ± 0.7 points) and good physical fitness (GPF) (N = 19; age: 14.5 ± 0.6 years; Height: 165.9 ± 5.8 cm; body mass: 58.9 ± 7.5 kg; body mass index: 21.5 ± 2.2 A.U.; ALPHA fitness score: 4.4 ± 0.3 points). The percentage of girl students assigned to each group (PPF and GPF) was similar (approximately 58.1%).

All participants and their parents were informed in writing and verbally of the purpose and procedures of the investigation, and the parents of the participants provided a signed informed consent before the start of the study. The participants were free to leave the activity without being required to provide any kind of explanation and without being penalised if they left the study. The study was approved by the Ethics Committee of Clinical Research at the Hospital Complex in Toledo (Spain) (number 721/21) according to the principles of the latest version of the Declaration of Helsinki [18].

### 2.2. Experimental Design

A cross-sectional and correlative design was used in this study. Each student was analysed during their participation in a single simulated ultimate frisbee match. The duration of the matches was 20 min divided into two halves of 10 min with 5 min rest between them. The study was conducted over a five-week period (February–March) and under the same experimental conditions (temperature ranged: 12 to 18 °C; wind range: 6 to 8 km·h^−1^). The matches were in the 4-a-side game modality and played on a field with dimensions of 20 × 40 m. In the previous weeks, the players were familiarised in two sessions with the basic technical–tactical aspects and rules of the ultimate frisbee game.

During the week, immediately before the study began, the physical condition of each participant was evaluated with the ALPHA fitness test [19]. The tests were applied by researchers (JP and AD) and the physical education teacher during a class, after a familiarisation session was carried out on a different day. The marks obtained by the participants were evaluated according to their age and gender, and rated from 0 to 5 points, 5 being the highest qualification. All tests were performed twice, and the best performance was chosen and expressed as a 0–5 mark [4]. The tests were as follows: cardiorespiratory fitness was assessed with the 20 m shuttle run test, agility was evaluated with the 4 × 10 m speed–agility test, lower-body explosive strength was measured with the broad jump test (standing long jump) and maximum handgrip strength was assessed with the handgrip strength test. The order for the test battery was:

The maximal handgrip strength was obtained using a hand dynamometer with an adjustable grip (TKK 5401 Grip D, Takey, Tokyo, Japan).

For the broad jump test, a tape measure was used to measure the maximum horizontal distance jumped. The student stood behind a line marked on the ground with feet together. A 2-foot take-off and landing was performed, with an arm swing and bend of the knees to provide forward drive. The subject attempted to jump as far as possible, landing on both feet without falling backwards. Each jump was measured for distance.

For the agility test or 10 × 5 m shuttle run test, the subject was required to run back and forth as fast as possible 10 times, along a 5 m course. The time to complete the agility test was recorded.

For the cardiorespiratory fitness test, an indirect and submaximal 20 m shuttle run field test performed until exhaustion was used to assess cardiorespiratory fitness. The running pace was marked by a beep. Initial speed was 8.5 km·h^−1^, with subsequent increases of 0.5 km·h^−1^ at 1 min intervals, called stages. Subjects had to run between the 20 m lines keeping time to when the beeps were heard. The test ended when the subject stopped due to fatigue or when they could not reach the line in time with the beep. Aerobic capacity was measured through maximum oxygen consumption by VO_2max_, estimated from the Léger equation [20]: ${\mathrm{VO}}_{2\max}\;\left(\mathrm{ml}\cdot {\mathrm{kg}}^{-1}\cdot \min^{-1}\right)=31.025+3.238+S-3.248\;\cdot A+0.1536\;\cdot S\;\cdot A$; where S is the speed (in km·h^−^^1^) of the last complete stage and A is age (in years) of the participant.

### 2.3. Match Analysis

Physical variables during the ultimate frisbee matches were analysed using a 15 Hz GPS (Spi HPU, GPSport^®^, Canberra, Australia). This system required players to wear a small article of clothing designed to carry the GPS device during the game. Validity of the GPS system has been reported in previous research [21,22]. In addition, GPS devices have been previously used to assess students’ physical performance during physical education lessons [23,24]. The distances covered in the ultimate frisbee match were estimated using Team AMS software version 7 (GPSport^®^, Canberra, Australia) and were presented in km·h^−1^. The speed zones were based on previous studies carried out on adolescents [21]: standing/walking or Zone 1: 0–0.4 km·h^−1^; walking or Zone 2: 0.5–3 km·h^−1^; jogging or Zone 3: 3.1–8 km·h^−1^; running or Zone 4: 8.1–13 km·h^−1^; high-speed running or Zone 5: 13.1–18 km·h^−1^; sprinting or Zone 6: >18 km·h^−1^. The number of accelerations and decelerations was also assessed [25].

Physiological demands were measured by analysing the time spent in each of the heart rate zones and the time above 80% intensity. Heart rates were monitored with short-range telemetry every 5 s during the matches (GPS Elite, GPSport^®^, Canberra, Australia). Maximal theorical heart rate was calculated using the formula proposed by Tanaka et al. [26] and was used to establish the intensity zones: Zone 1 (<60% of maximal heart rate (HRmax)), Zone 2 (60–70% HRmax), Zone 3 (70–80% HRmax), Zone 4 (80–90% HRmax), Zone 5 (90–95% HRmax), Zone 6 (95–100% HRmax). The percentage of spent time in relation to the duration of the game was calculated for each heart rate zone.

Two video cameras (JVC GZ-R315BEU Full HD video camera, JVC^®^, Tokyo, Japan) set diagonally, approximately 5 m behind the base line of each half-court and placed 2 m above the floor, were used to record each game action during the matches [27]. Participants wore jerseys differentiated by colours and numbers to facilitate video analysis. Technical–tactical skills were measured using the Game Performance Assessment Instrument adapted to invasive team sports [28]. The game actions were evaluated by ultimate frisbee coaches of the Spanish ultimate frisbee team and by researchers. In case of disagreement among the observers in the evaluation of the action, the observers replayed the specific action and discussed it until a final decision was reached. The technical and tactical actions were recorded with Longomatch software (Longomatch, ver. 0.20.8, Barcelona, Spain. Available online: http://longomatch.org (accessed on 1 June 2021)), which includes a template manager for categories allowing creation and modification of templates of up to 20 categories, into which the technical and tactical principles are introduced, establishing a name and colour that describes the corresponding category. The analysed technical variables were the number of passes (throw inside the reception area or not), attacking movements (movement with the aim of getting away from the defender or not), recoveries and defensive actions (putting pressure on the opponent or not) and offensive decision making (correctly selecting the player for passing the frisbee or not). All technical actions were coded as positive (successful) or negative (mistake). In addition, three indices were calculated: decision index (right decision related to total decisions); skill execution index (number of successful actions related to total actions) and player participation (sum of total actions). The level of agreement for the technical and tactical analysis was determined using the percentage of agreement between two repeated observations (from a pilot study) to provide an indication in the consistency of the data [28]. The percentages of exact agreements for both inter- and intra-reliability were 93%. This score is above the value suggested by Van der Mars [29] as suitable for a complex system.

### 2.4. Statistical Analysis

Data analysis was performed using the SPSS v 24.0 software (SPSS Inc., Chicago, IL, USA). Data were expressed as mean ± SD for the 43 secondary school students. The Shapiro–Wilk test was used to analyse normality in all variables. After the assumption of normality for all the variables (*p* > 0.05), Student’s t test for independent samples was used to establish the differences between the two groups (PPF and GPF). The magnitude of difference for pairwise comparisons between the PPF and GPF groups was quantified using the formula proposed by Cohen [30]. The magnitude of the effect size (ES) was interpreted using Cohen’s d [30]: an ES lower than 0.2 was considered to be small; an ES around 0.5 was considered to be medium and, an ES over 0.8 was considered to be large. The relationship between physical and technical–tactical variables was analysed with simple linear regression, from which the Pearson correlation coefficient was calculated. The criterion for significance was *p* ≤ 0.05 for all statistical tests.

## 3. Results

After the ALPHA fitness test battery was administered, the results of our study showed that 55.8% of the high school students had poor physical fitness in relation to their age (24 of 43 students). GPF students achieved higher values (GPF vs. PPF) in maximal hand grip strength (33.16 ± 8.65 vs. 27.92 ± 5.72 N; *p* = 0.022), standing broad jump (1.90 ± 0.32 vs. 1.53 ± 0.27 m; *p* < 0.001), cardiorespiratory fitness test (50.67 ± 6.05 vs. 43.02 ± 7.90 mL·kg^−1^·min^−1^; *p* = 0.001) and lower values for the agility test (10.27 ± 0.57 vs. 11.87 ± 1–30 s; *p* < 0.001).

**Figure 1:**
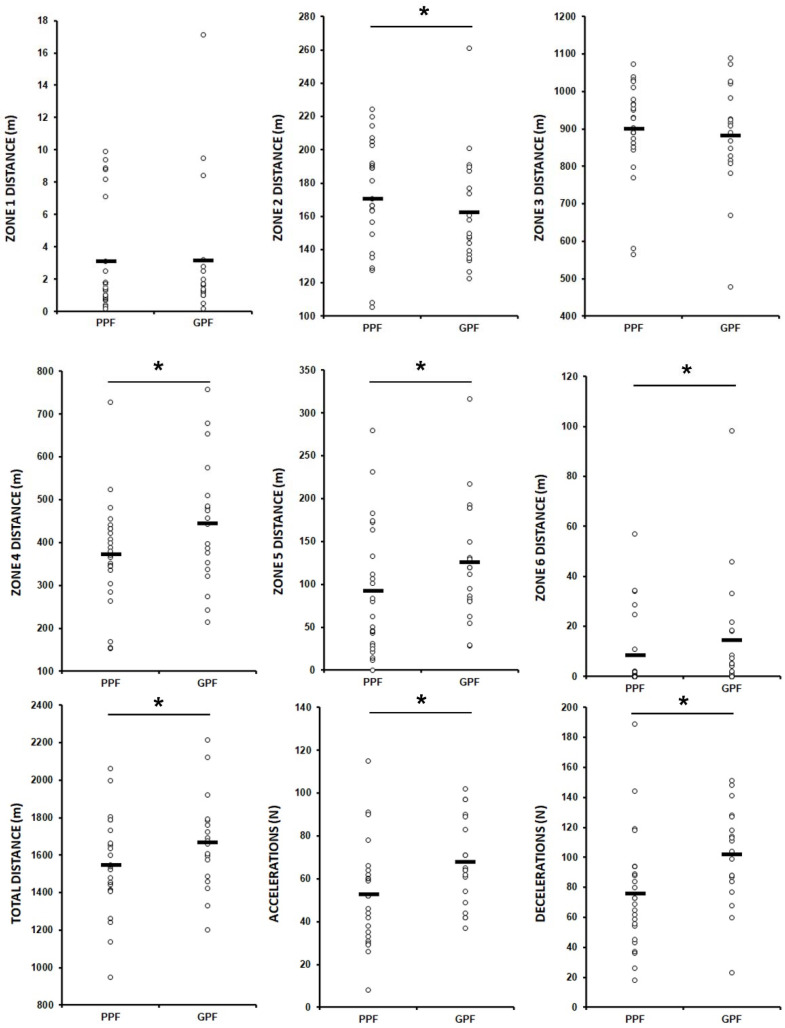
Physical performance during the ultimate frisbee match. PPF = poor physical fitness group, GPF = good physical fitness group, * = differences with *p* < 0.05 between PPF and GPF.

The physical variable results are presented in Figure 1. GPF students recorded a better physical performance during the ultimate frisbee game compared to PPF students showing more accelerations (67.58 ± 20.51 vs. 52.63 ± 24.07 accelerations; ES = 0.67; *p* = 0.037), decelerations (101.53 ± 32.21 vs. 75.63 ± 38.81 decelerations; ES = 0.73; *p* = 0.024) and total distance covered (1695.3 ± 248.6 vs. 1520.9 ± 239.3 m; ES = 0.71; *p* = 0.025). In this vein, the analysis of speed zones showed that the GPF group covered 23.7% more metres in the running zone or Zone 4 (ES = 0.65; *p* = 0.039), 66.7% in the high-speed running zone or Zone 5 (ES = 0.77; *p* = 0.015) and 282.4% in the sprinting zone or Zone 6 (ES = 0.70; *p* = 0.022) than the PPF group. In addition, the distance covered in the walking zone or Zone 2 was 11.8% lower for the GPF students than the PPF students (ES = 0.64; *p* = 0.046). No differences were found for Zone 1 (*p* = 0.640) and Zone 3 (*p* = 0.912). Finally, we did not find differences for any heart rate zone between GPF and PPF (*p* > 0.669) (Table 1).

The technical–tactical variable results are presented in Table 2. The number of passes for GPF students was 75.0% greater than for the PPF group (ES = 0.66; *p* = 0.019). The offensive decision making (number of correct selections of the player for passing the frisbee) was 86.7% greater for the GPF group than the PPF group (ES = 0.82; *p* = 0.009) and player participation was also higher (27.2% of difference; ES = 0.72; *p* = 0.046) for the GPF group than the PPF group. No differences were observed for the number of attacking movements (*p* = 0.275), the number of recoveries and defensive actions (*p* = 0.770), the decision index (*p* = 0.628) or the skill execution index (*p* = 0.493).

A positive correlation was found between participation and distance covered in Zone 3 (r = 0.368; *p* = 0.035), Zone 4 (r = 0.532; *p* = 0.001), Zone 5 (r = 0.579; *p* < 0.001) and Zone 6 (r = 0.388; *p* = 0.026), average speed (r = 0.407; *p* = 0.029), total distance (r = 0.501; *p* = 0.003) and total accelerations (r = 0.370; *p* = 0.034). In addition, the skill execution index showed a positive correlation with distance covered in Zone 3 (r = 0.375; *p* = 0.031) and Zone 4 (r = 0.380; *p* = 0.029).

## 4. Discussion

Performance in team sports is associated with different physical, mental, physiological, technical and tactical components. The main purpose of this research was to determine whether the physical fitness of secondary school students affects their performance during a simulated ultimate frisbee match. The results of this study showed that students from the GPF group covered more meters in Zone 4 (running), Zone 5 (high-speed running) and Zone 6 (sprinting) during a competitive match than the PPF group students. After correlational analysis, positive relationships were described between player participation and distance covered in jogging, running, running at high speed and sprinting. Therefore, higher distance covered in speed zones from jogging to sprinting efforts could increase the level of participation, or total number of actions, of the students during the game, which could emphasis the role of physical fitness in novice sport participants performance. Finally, no differences were described in the qualitative variables, decision index and skill execution index, between GPF and PPF students, but GPF students achieved greater participation in the game. This could be due to GPF students making more mistakes when they moved at high velocities because they did not have enough sport knowledge. Physical education teachers should focus their attention on students with low levels of physical fitness so that they can improve their physical capacities, which will thus enable students to better harness opportunities provided by physical education classes.

The analysis of running intensity defines the pace of each player during the game and the metres covered in the high intensity running zones are associated with the best sports performance [31]. Previous studies have described a positive relationship between individual physical fitness and running performance in team sports for elite, sub-elite, recreational and school participants [16,17,32]. In this vein, we found that the GPF group covered more metres in Zone 4 (running), Zone 5 (high-speed running) and Zone 6 (sprinting) than the PPF students. In addition, few students from the PPF group could run in the sprinting zone compared to the GPF group (9 vs. 15 students), so individual fitness seems to be an important factor for physical performance. Accordingly, Fairclough and Stratton [7] stated that high-ability students spend more time during physical education sessions in MVPA zones than average and low-ability students.

High physical fitness provides an advantage in activities where the physical and skill demands are greater, such as team sports [33]. The largest differences in physical performance between the GPF and PPF group in our study were described in Zones 5 and 6. The high intensity efforts of an intermittent nature are the most demanded in team sports [13]. From this perspective, we can consider that good physical fitness is necessary to respond to the variety of efforts that are needed during an ultimate frisbee match, so low values of physical fitness would be a negative aspect regarding obtaining a good sports performance. The intermittent efforts carried out during the game could be an effective training stimulus to achieve physiological adaptations [13]. Finally, the intensity of the activity during physical education sessions could help the students to achieve the recommendations of daily physical activity [3], so it is important to focus attention on students with a poor level of physical fitness to improve their physical performance during invasion games.

In team sports, an optimal level of physical fitness needs to be integrated with the execution of basic technical–tactical skills to achieve a good level of performance. In our study, the GPF students performed more passes and offensive decision making, their game participation was also higher compared to the PPF students. The physical fitness level correlated positively with passing accuracy in junior football players [34] and with the index ratio (rebounds, assists, points) in basketball [35]. In addition, the football players with a high work/rest ratio made more dribbles and shots during an 8-a-side football match [36]. Similarly, we found a positive correlation between physical performance and player participation during the game. Experience in the game could help players with low physical fitness to achieve a similar game performance to that of players with a good physical fitness level [17,37] but in our study, the schoolchildren were novices in ultimate frisbee so their experience did not compensate for their physical fitness deficit. In this sense, it is possible that the skill execution index was affected by the experience of the players because good executions were only associated with the jogging and running speed zones. Therefore, future studies are warranted to corroborate the association between the physical and technical–tactical performance of high school students in sports in which they are experts.

Our study has several limitations: (1) The competition model was mixed-sex, so the results may not describe the performance of all the students during single-sex competitions. New studies are necessary to advance the knowledge of schoolchildren’s behaviours under mixed-sex and single-sex competition conditions and to analyse which competition framework is the best to promote the physical and game profile of the students. (2) Performance in team sports is multifactorial and our study only analysed physical, physiological demands and game performance. (3) The students were novices in ultimate frisbee, so the results should be corroborated with expert players to establish the level of sporting efficiency reached by the novice students. (4) Finally, the sample size of 43 secondary students was rather small, further studies with large cohorts are essential to corroborate these findings.

### Practical Applications

The results of the study showed that schoolchildren with better physical fitness achieved better scores in the physical demands of match and game performance during ultimate frisbee matches. The level of physical fitness could impact the sports performance of secondary school students during the game of ultimate frisbee. Therefore, during the teaching of new sports, secondary education teachers should design tasks that favor a high level of participation of all students, regardless of their physical fitness level. In addition, the use of recreational games in simulated competitive conditions is a framework that can help students to improve physical fitness and performance in sports, as the physical, physiological and technical–tactical demands are high.

## 5. Conclusions

To the best of the authors’ knowledge, this is the first study to examine the effect of physical fitness on physical and technical–tactical performance of schoolchildren with no previous experience in ultimate frisbee. The findings indicate that the students with better physical fitness covered more metres in the high intensity speed zones, with the greatest difference in the sprint zone. The students with better physical fitness also made more passes, offensive actions and had a greater participation in the game. The numerous correlations found in our study suggest that higher physical fitness positively impacts school children’s outcomes in both running demands and technical–tactical performance during ultimate frisbee matches.

## Figures and Tables

**Table 1:** Percentage of time in each heart rate zone during the ultimate frisbee match.

	Poor Physical Fitness	Good Physical Fitness	*p*	Δ	Confidence Interval of 95%
Zone 1 (<60% HRmax)	13.28 ± 7.38	14.48 ± 5.23	0.687	1.20	−4.95 to 7.32
Zone 2 (60–70% HRmax)	14.86 ± 5.52	14.31 ± 7.18	0.848	0.55	−6.51 to 5.41
Zone 3 (70–80% HRmax)	10.16 ± 5.58	9.62 ± 7.71	0.858	0.54	−6.81 to 5.73
Zone 4 (80–90% HRmax)	19.59 ± 8.37	17.81 ± 9.96	0.669	1.78	−10.39 to 6.83
Zone 5 (90–95% HRmax)	28.85 ± 12.80	29.74 ± 16.72	0.893	0.90	−12.95 to 14.75
Zone 6 (95–100% HRmax)	13.03 ± 13.21	15.14 ±16.56	0.754	2.12	−11.85 to 16.09

**Table 2:** Technical–tactical performance variables.

	Poor Physical Fitness	Good Physical Fitness	*p*	Δ	Confidence Interval of 95%
Passes (N)	13.50 ± 11.14	23.63 ± 16.01	0.019	10.13	1.76 to 18.50
Attacking moves (N)	9.67 ± 8.64	12.63 ± 8.85	0.275	2.96	−2.44 to 8.38
Defensive actions (N)	12.50 ± 8.66	13.26 ± 8.13	0.770	0.76	−4.47 to 5.99
Offensive decision-making (N)	11.42 ±9.32	21.32 ± 14.42	0.009	9.90	2.56 to 17.24
Decision index (A.U.)	0.79 ± 0.10	0.81 ± 0.12	0.628	0.02	−0.06 to 0.10
Skill execution index (A.U.)	0.75 ± 0.09	0.77 ± 0.09	0.493	0.02	−0.04 to 0.09
Player participation (N)	80.41 ± 23.39	102.25 ± 35.96	0.046	21.84	0.43 to 43.25

## Data Availability

The data presented in this study are available on request from the corresponding author. The data are not publicly available due to restrictions of the subjects’ agreement.

## References

[1] Sibley B.A., Etnier J.L. (2003). The Relationship between Physical Activity and Cognition in Children: A Meta-Analysis. Pediatr. Exerc. Sci..

[2] Jiménez-Maldonado A., Rentería I., García-Suárez P.C., Moncada-Jiménez J., Freire-Royes L.F. (2018). The Impact of High-Intensity Interval Training on Brain Derived Neurotrophic Factor in Brain: A Mini-Review. Front. Neurosci..

[3] Hollis J.L., Sutherland R., Williams A.J., Campbell E., Nathan N., Wolfenden L., Morgan P.J., Lubans D.R., Gillham K., Wiggers J. (2017). A systematic review and meta-analysis of moderate-to-vigorous physical activity levels in secondary school physical education lessons. Int. J. Behav. Nutr. Phys. Act..

[4] Ruiz J., Romero V.E., Piñero J.C., Artero E., Ortega F., García M.C., Pavón D.J., Chillón P., Rejón M.J.G., Mora J. (2011). Batería ALPHA-Fitness: Test de campo para la evaluación de la condición física relacionada con la salud en niños y adolescentes. Nutr. Hosp..

[5] Fairclough S., Stratton G. (2005). Physical Activity Levels in Middle and High School Physical Education: A Review. Pediatric Exerc. Sci..

[6] Spessato B.C., Gabbard C., Valentini N.C. (2013). The Role of Motor Competence and Body Mass Index in Children’s Activity Levels in Physical Education Classes. J. Teach. Phys. Educ..

[7] Fairclough S., Stratton G. (2005). ‘Physical education makes you fit and healthy’. Physical education’s contribution to young people’s physical activity levels. Health Educ. Res..

[8] Harvey S., Garcia López L. (2017). Objectively Measured Physical Activity of Different Lesson Contexts. J. Phys. Educ. Sport.

[9] Brusseau T.A., Kulinna P.H. (2015). An Examination of Four Traditional School Physical Activity Models on Children’s Step Counts and MVPA. Res. Q. Exerc. Sport.

[10] Hastie P., Trost S. (2002). Student Physical Activity Levels during a Season of Sport Education. Pediatr. Exerc. Sci..

[11] Krustrup P., Mohr M. (2015). Physical Demands in Competitive Ultimate Frisbee. J. Strength Cond. Res..

[12] Hannon J.C. (2008). Physical activity levels of overweight and nonoverweight high school students during physical education classes. J. Sch. Health.

[13] Gabbett T., Jenkins D., Abernethy B. (2009). Game-Based Training for Improving Skill and Physical Fitness in Team Sport Athletes. Int. J. Sports Sci. Coach..

[14] Britton Ú., Belton S., Issartel J. (2019). Small fish, big pond: The role of health-related fitness and perceived athletic competence in mediating the physical activity-motor competence relationship during the transition from primary to secondary school. J. Sports Sci..

[15] Robinson L.E., Stodden D.F., Barnett L.M., Lopes V.P., Logan S.W., Rodrigues L.P., D’Hondt E. (2015). Motor Competence and its Effect on Positive Developmental Trajectories of Health. Sports Med..

[16] Miller A., Eather N., Lubans D.R., Duncan M. (2019). Associations of object control motor skill proficiency, game play competence, physical activity and cardiorespiratory fitness among primary school children. J. Sports Sci..

[17] Tissera K., Naughton G., Gabbett T., Krause L., Moresi M., Benson A. (2019). Sex Differences in Physical Fitness Characteristics and Match-Play Demands in Adolescent Netball: Should Male and Female Adolescents Co-compete in Netball?. J. Strength Cond. Res..

[18] World Medical Association (2013). World Medical Association Declaration of Helsinki: Ethical principles for medical research involving human subjects. JAMA.

[19] Ruiz J.R., Castro-Piñero J., España-Romero V., Artero E.G., Ortega F.B., Cuenca M.M., Jimenez-Pavon D., Chillon P., Girela-Rejon M.J., Mora J. (2011). Field-based fitness assessment in young people: The ALPHA health-related fitness test battery for children and adolescents. Br. J. Sports Med..

[20] Leger L.A., Mercier D., Gadoury C., Lambert J. (1988). The multistage 20 metre shuttle run test for aerobic fitness. Journal of Sports Sciences..

[21] Castagna C., Impellizzeri F., Cecchini E., Rampinini E., Barbero J. (2009). Effects of intermittent-endurance fitness on match performance in young male soccer players. J. Strength Cond. Res..

[22] Coutts A.J., Duffield R. (2010). Validity and reliability of GPS devices for measuring movement demands of team sports. J. Sci. Med. Sport.

[23] Gonzalez-Espinosa S., Antunez A., Feu S., Ibanez S.J. (2020). Monitoring the External and Internal Load Under 2 Teaching Methodologies. J. Strength Cond. Res..

[24] García-Ceberino J.M., Antúnez A., Feu S., Ibáñez S.J. (2020). Quantification of Internal and External Load in School Football According to Gender and Teaching Methodology. Int. J. Environ. Res. Public Health.

[25] Couderc A., Gabbett T., Piscione J., Robineau J., Peeters A., Igarza G., Thomas C., Hanon C., Lacome M. (2019). Repeated High-Intensity Effort Activity in International Male Rugby Sevens. J. Strength Cond. Res..

[26] Tanaka H., Monahan K.D., Seals D.R. (2001). Age-predicted maximal heart rate revisited. J. Am. Coll. Cardiol.

[27] Pérez-López A., Salinero J.J., Abian-Vicen J., Valadés D., Lara B., Hernandez C., Areces F., González C., Del Coso J. (2014). Caffeinated Energy Drinks Improve Volleyball Performance in Elite Female Players. Med. Sci. Sports Exerc..

[28] Mitchell S.A., Oslin J.L., Griffin L.L. (2006). Teaching Sport Concepts and Skills: A tactical Games Approach.

[29] Van der Mars H., Darst P.W., Zakrajsek D.B., Mancini V.H. (1989). Observer reliability: Issues and procedures. Analyzing Physical Education and Sport Instruction.

[30] Cohen J. (1988). Statistical Power Analysis for the Behavioral Sciences.

[31] Ross A., Gill N., Cronin J., Malcata R. (2015). The relationship between physical characteristics and match performance in rugby sevens. Eur. J. Sport Sci..

[32] Aquino R., Carling C., Maia J., Vieira L.H.P., Wilson R.S., Smith N., Almeida R., Gonçalves L.G.C., Kalva-Filho C.A., Garganta J. (2020). Relationships between running demands in soccer match-play, anthropometric, and physical fitness characteristics: A systematic review. Int. J. Perform. Anal. Sport.

[33] Hardy L.L., Reinten-Reynolds T., Espinel P., Zask A., Okely A.D. (2012). Prevalence and Correlates of Low Fundamental Movement Skill Competency in Children. Pediatrics.

[34] Impellizzeri F., Rampinini E., Maffiuletti N., Castagna C., Bizzini M., Wisløff U. (2008). Effects of aerobic training on the exercise-induced decline in short-passing ability in junior soccer players. Appl. Physiol. Nutr. Metab..

[35] Garcia-Gil M., Torres-Unda J., Esain I., Duñabeitia I., Gil S.M., Gil J., Irazusta J. (2018). Anthropometric Parameters, Age, and Agility as Performance Predictors in Elite Female Basketball Players. J. Strength Cond. Res..

[36] Bravo-Sánchez A., Abián-Vicén J., Abián P. (2017). Analysis of the physical and technical differences between 7-a-side and 8-a-side game modalities in official under 12 soccer matches. Int. J. Perform. Anal. Sport.

[37] Modric T., Versic S., Sekulic D. (2021). Does aerobic performance define match running performance among professional soccer players? A position-specific analysis. Res. Sports Med..

